# Cooperative impact of thiazolidinedione and fatty acid synthase on human osteogenesis

**DOI:** 10.18632/aging.101916

**Published:** 2019-04-20

**Authors:** Ching-Yun Chen, Kuo-Yun Tseng, Zhe-Hong Wong, Ya-Ping Chen, Ting-Yu Chen, Hsuan-Ying Chen, Zih-Ying Chen, Feng-Huei Lin, Hung-Ming Wu, Shankung Lin

**Affiliations:** 1 Institute of Biomedical Engineering, College of Medicine and College of Engineering, National Taiwan University, Taiwan, Republic of China; 2 Institute of Biomedical Engineering and Nanomedicine, National Health Research Institutes, Taiwan, Republic of China; 3 Institute of Infectious Diseases and Vaccinology, National Health Research Institutes, Taiwan, Republic of China; 4 Department of Orthopedics, National Taiwan University Hospital, Hsin-chu Branch, Taiwan, Republic of China; 5 Inflammation Research and Drug Development Center, Taiwan, Republic of China; 6 Department of Neurology, Changhua Christian Hospital, Taiwan, Republic of China; 7 Graduate Institute of Acupuncture Science, China Medical University, Taiwan, Republic of China; 8 Graduate Institute of Biomedical Sciences, China Medical University, Taiwan, Republic of China; * Equal contribution

**Keywords:** thiazolidinedione, bioreactor, 3-dimentional culture, bone-like tissues, autologous cell therapy

## Abstract

Previous, we found that the small molecules capable of inhibiting the expression and the pro-adipogenic activity of ZNF521 might improve the osteogenic performance of aging human bone marrow MSCs (bmMSCs), and that fatty acid synthase (FASN) was a critical effector of ZNF521’s pro-adipogenic activity. Here, by characterizing the netoglitazone (MCC-555), one of the thiazolidinediones known as adipogenic enhancers, as an inhibitor of ZNF521 expression, we found that MCC-555 indeed also harbored pro-osteoblastic effect. Investigation revealed that MCC-555 might function as a GSK3β inhibitor to promote osteoblastogenesis and bone formation. Importantly, combination of MCC-555 with FASN knockdown, but not with GW9662 (a PPARγ2 antagonist), blocked the pro-adipogenic but retained the pro-osteoblastic effect of MCC-555. Using a 3-dimentional culture system, we showed that MCC-555 facilitated the FASN-knockdown of aging human bmMSCs to form cell clusters in scaffolds, and to promote osteoblastic differentiation and biomineralization in cell clusters. These data indicated that MCC-555 promoted bmMSCs to produce bone-like tissues. Our data narrate a thiazolidinedione-based novel strategy to improve the osteogenic performance of aging bmMSCs to support the application of autologous aging bmMSCs in cell therapy and in producing bone-like tissues for repairing bone injury in the elderly.

## INTRODUCTION

Osteoporosis is an aging-associated disease featured by severe bone loss and increased bone fragility, which makes the elderly peoples prone to fracture fall. Due to the impairment in bone formation activity, the elderly patients tend to suffer sustained bone injury, which debilitates the patients and could be even fatal. It is predictable that the global medical demands for the prevention of osteoporotic bone loss and the treatment of osteoporotic fracture will increase as the longevity increases around the world.

Current pharmacological treatment of osteoporotic bone loss includes inhibition of bone resorption and stimulation of bone formation using hormones, bisphosphonates, antibodies, protein inhibitors, nutritional supplements, etc. [1–3]. For example, oestrogen, bisphosphonates, and denosumab (a RANKL antibody) are used to inhibit bone resorption, whereas parathyroid hormone (PTH) and teriparatide (a PTH derivative) are used to increase bone formation. These agents are not without undesired side effects. The inhibitors of bone resorption effectively decrease bone loss, however, they also block the removal of damaged bones. As a result, bone mass is maintained at the expense of bone quality and strength. PTH and its derivatives may also stimulate bone resorption when administered continuously and result in net bone loss. The conventional way to treat osteoporotic fracture is surgery. But due to the poor bone formation activity in the elderly people, it takes more time for the elderly patients to recover from the injury and surgery, and after that the poor bone quality remains. It can be concluded, in view of the effectiveness and limit of current strategies, that improving bone formation activity and allowing the removal of damaged bones is a better way to decrease bone loss and improve bone strength for the treatment of osteoporosis and osteoporotic fracture.

The role of the canonical Wnt/β-catenin pathway in regulating bone formation has been well documented [reviewed in refs. 4, 5]. The binding of Wnt ligand to the frizzled receptor results in the serine 9 (Ser9)-phosphorylation of glycogen synthase kinase 3β (GSK3β), which inactivates GSK3β and prevents it from phosphorylating β-catenin at the Ser33/37/Thr41 sites, which, in turn, inhibits the ubiquitination and degradation of β-catenin in the cytoplasm [6]. The accumulated β-catenin translocates into nucleus and complexes with coactivators to transactivate genes involved in osteoblastogenesis and bone formation. GSK3β is, therefore, a therapeutic target for stimulating bone formation. GSK3β inhibitors such as lithium, 603281-31-8, and AR28 have been tested for increasing bone mass [7].

Several lines of evidences indicate that bone marrow mesenchymal stem cells (bmMSCs) is a critical component in the aging-related loss of bone formation activity. In humans, aging is associated with insufficient osteoblasts [8–10]. In aged rats, the bone marrows are more adipogenic and less osteoblastogenic than those of young rats [11]. Moreover, aging bmMSCs exhibit an increase in the commitment to adipogenic lineage and a decrease in the commitment to osteoblastic lineage [12–15], and zinc finger factor 521 (ZNF521) has been proposed as a critical regulator of these processes [16, 17]. In paticular, ZNF521 knockdown enhances osteoblastic differentiation of human bmMSCs [17], implying that the small molecules which inhibit the expression and/or the pro-adipogenic activity of ZNF521 could be the candidates to be developed into drugs to increase the osteogenic performance of aging bmMSCs.

Thiazolidinediones (TZDs) are potent antidiabetic agents, functioning mainly through their agonistic effect on the peroxisome proliferator-activated receptor γ (PPARγ) [18, 19]. However, PPARγ is not only the major transcriptional activator of adipogenesis, but also a repressor of osteoblastogenesis [20]. Therefore, the antidiabetic effect of TZDs is confronted by undesired bone loss [21–24]. These findings have stereotyped TZDs as adipogenic enhancers. Extensive efforts have been made to synthesize new TZDs with safer bone effect [reviewed in ref. 25], which may have slow down the investigation regarding the panoramic effect of TZD on cell fate. Interestingly, our previous studies showed that troglitazone was an inhibitor of ZNF521 expression [17]. Little is known if the inhibitory effect on ZNF521 expression is an idiosyncratic effect of troglitazone or a class effect of TZDs, nor is it clear if TZDs elicit ZNF521 inhibition and also harbor pro-osteogenic effect. Clarification on these issues may modify our perspective on the biological effect of TZDs. On the other hand, we have recently reported that ZNF521 promoted adipogenesis by enhancing expression of fatty acid synthase (FASN); FASN knockdown prevented ZNF521 from enhancing adipogenesis [26]. This finding suggests that FASN is an important effector of ZNF521 and, therefore, is a potential target for the inhibition of ZNF521’s pro-adipogenic activity. It is then important to examine if the combination of TZD and FASN inhibition exhibits differential effects on the adipogenic and osteoblastic differentiation as those noted in cells with ZNF521 knockdown. In this study, we have used 2-dimentional cultures to investigate the enhancing effect of netoglitazone (MCC-555) on the osteoblastic differentiation of mouse C3H10T1/2 cells and aging human bmMSCs in the background of FASN knockdown. We have also used a bioreactor system as a platform to mimic *in vivo* condition to further prove the enhancing effect of MCC-555 plus FASN knockdown on the bone-forming capability of aging human bmMSCs growing in porous 3-dimentional calcium-alginate scaffolds. This bioreactor system has been shown to successfully support human osteoblasts and bmMSCs to produce bone-like tissues *in vitro* [27–29].

## RESULTS

### MCC-555 enhanced adipogenic and osteoblastic differentiation of C3H10T1/2 cells

**Figure 1:**
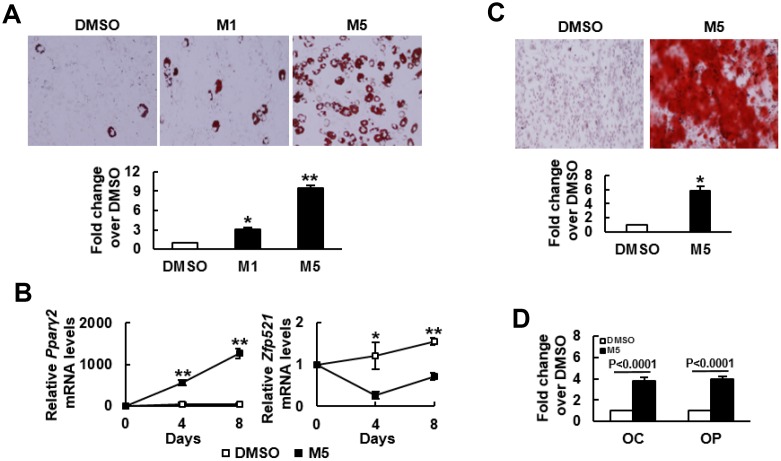
**MCC-555 enhanced adipogenic and osteoblastic differentiation.** (**A**) Adipogenic induction. Confluent C3H10T1/2 cells under adipogenic induction were co-treated with either DMSO (DMSO) or with 1 μM (M1) or 5 μM (M5) MCC-555 in the first 3 days. Cells were stained with Oil Red O at the 8^th^ day. Representative photos are shown. The stains were quantitated, and the signals of the TZD-treated cells were compared to that of the untreated cells (to which a value of 1 was assigned). ^*^, P<10^-4^, ^**^, P<10^-5^ versus DMSO control. (**B**) RT-qPCR analyses. C3H10T1/2 cells were treated with MCC-555 as described in (**A**). Total RNAs were isolated at the times as indicated, and the kinetic expression of *Pparγ2* and *Zfp521* mRNAs were shown. ^*^, P<0.05, ^**^, P<0.0005 versus counterpart DMSO-treated controls. (**C**) Osteoblastic induction. Confluent C3H10T1/2 cells were subjected to osteoblastic induction with the co-treatment of either DMSO or 5 μM MCC-555 (M5). Cells were stained with Alizarin Red S at the 28^th^ day. Representative photos are shown. The stains were quantitated, and the signals of the MCC-555-treated cells were compared to that of the DMSO-treated cells (to which a value of 1 was assigned). ^*^, P<10^-8^ versus DMSO control. (**D**) RT-qPCR analyses. Cells treated with 5 μM MCC-555 (M5) as described in (**C**) were harvested 29 days post-induction, and subjected to RT-qPCR analyses for *osteocalcin* (OC) and *osteopontin* (OP) mRNAs. Data represent the mean ± S.D. from three experiments.

We induced C3H10T1/2 cells to undergo adipogenic differentiation in the presence or absence of MCC-555. MCC-555 (1 μM and 5 μM) dose-dependently enhanced lipid droplet formation, as evidenced by Oil Red O staining at day 8 post-induction (Figure 1A). Therefore, 5 μM MCC-555 was selected for use in all further studies. RT-qPCR analyses showed that adipogenic induction induced largely 50 fold increase in the expression of *Pparγ2* mRNA in the DMSO-treated cells at day 4 and day 8 after induction, whereas MCC-555 co-treatment induced approximately 500 to 1300 fold increase (Figure 1B). In the expression of *Zfp521* mRNA, adipogenic induction induced approximately 20% and 54% increase in the DMSO-treated cells at day 4 and day 8 after induction, but conversely, MCC-555 co-treatment caused approximately 70% and 30% decrease, respectively (Figure 1B). This set of data led us to examine if MCC-555 was able to enhance osteoblastic differentiation. We induced C3H10T1/2 cells to the osteoblastic lineage in the presence or absence of MCC-555. As evidenced by Alizarin Red S staining at day 28 post-induction, MCC-555 significantly enhanced calcium precipitation compared with DMSO control (Figure 1C). Parallel RT-qPCR analyses showed that MCC-555 induced approximately 4 fold increase in the expression of two osteoblast markers, osteocalcin and osteopontin, at day 29 post-induction (Figure 1D). Taken together, our data revealed that MCC-555, well recognized as an adipogenic stimulator, was shown also, in fact, an enhancer of osteoblastic differentiation. This duel enhancing effect was also seen with troglitazone (data not shown), supporting it as a class effect of TZDs.

### MCC-555 enhanced nuclear translocation of β-catenin and inhibited GSK3β activity

**Figure 2:**
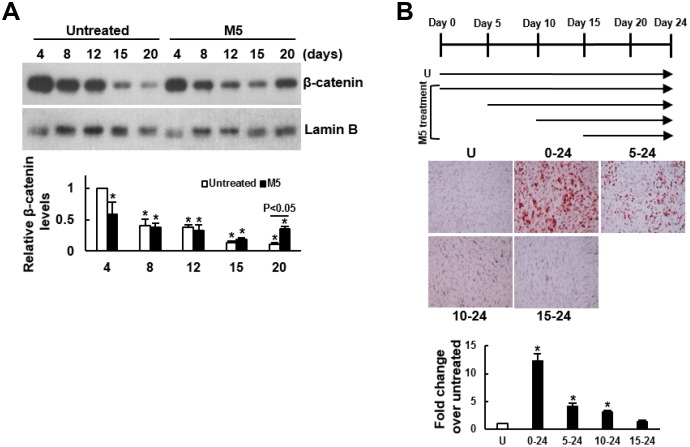
**Delineation of the mechanism underlying the enhancing effect of MCC-555 on the osteoblastic differentiation of C3H10T1/2 cells.** (**A**) Western blot analyses. Confluent C3H10T1/2 cells were induced to undergo osteoblastic differentiation with either vehicle (Untreated) or MCC-555 co-treatment (5 μM, M5). Cells were harvested at the times as indicated for the preparation of nuclear fractions. β-catenin and Lamin B (as a normalizer) were detected and quantitated. Relative β-catenin levels were calculated by comparing the normalized signals of MCC-555-treated cells to that of the untreated cells at day 4 (to which a value of 1 was assigned). Data represent the mean ± S.D. from three experiments. One-way ANOVA plus Scheffe’s post hoc tests were used to analyze the differences. ^*^, P<0.05 versus the untreated cells of day 4. Student’s t-test was used to analyze the difference between the untreated and M5-treated cells at day 20. (**B**) Osteoblastic induction. Confluent C3H10T1/2 cells were induced to undergo osteoblastic differentiation. Five μM MCC-555 (M5) was added at days 0, 5, 10, and 15 as indicated in the schematic presentation (upper), and was changed along with the media every 3 days. Cells were stained with Alizarin Red S at the 24^th^ day. Representative photos are shown (lower). The stains were quantitated, and the signals of the MCC-555-treated cells were compared to that of the vehicle-untreated cells (U) (to which a value of 1 was assigned). One-way ANOVA plus Scheffe’s post hoc tests were used to analyze the differences. ^*^, P<0.05 versus the untreated cells.

To explore the potential mechanism underlying MCC-555’s pro-osteogenic effect, we examined if MCC-555 increased the nuclear levels of β-catenin given that the translocation of β-catenin from cytoplasm to nucleus promotes osteoblastogenesis. We induced C3H10T1/2 cells to the osteoblastic lineage with or without MCC-555 co-treatment for 4, 8, 12, 15, and 20 days, and examined the nuclear β-catenin levels. Western blot analyses showed that in the control (DMSO-treated) cells, the nuclear β-catenin levels decreased with time, whereas in the MCC-555-treated cells, the β-catenin levels also decreased with time till day 15, and then increased at day 20 with the level significantly higher than that of the counterpart control cells (P<0.05) (Figure 2A). Next, we induced C3H10T1/2 cells to the osteoblastic lineage, and co-treated cells with MCC-555 at different periods of time (Figure 2B, upper panel). Alizarin Red S staining performed 24 days post-induction showed that MCC-555 co-treatment from day 0 to day 24 resulted in approximately 11 fold (P<0.01) increase over the untreated control in calcium precipitation, whereas the MCC-555-induced calcium precipitation activity was only 4 (P<0.05) and 3 (P<0.05) fold of the untreated control when MCC-555 was added at day 5 and day 10 post-induction, respectively (Figure 2B, lower panel). MCC-555 added at day 15 post-induction was not able to enhance calcium precipitation. Together, these data suggested that nuclear translocation of β-catenin might be involved in the pro-osteogenic effect of MCC-555, and that the first 5 days was critical for MCC-555 to promote osteoblastic differentiation.

**Figure 3:**
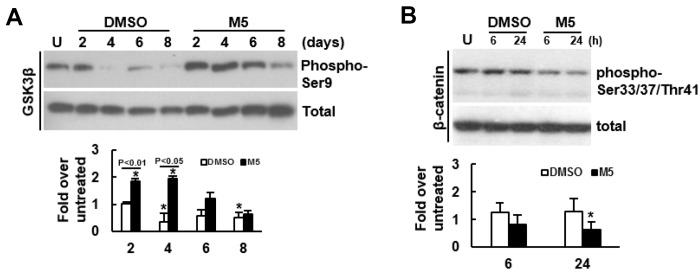
**Effect of MCC-555 on the Wnt/β-catenin signaling of C3H10T1/2 cells.** (**A**) Western blot analyses. Confluent C3H10T1/2 cells were induced to undergo osteoblastic differentiation for 2, 4, 6, and 8 days with either vehicle (DMSO) or MCC-555 co-treatment. The signals of Ser9-phosphorylated GSK3β were quantitated and normalized to total GSK3β. All the normalized signals were compared to that of the untreated control (U) (to which a value of 1 was assigned). Data represent the mean ± S.D. from three experiments. One-way ANOVA plus Scheffe’s post hoc tests were used to analyze the differences. ^*^, P<0.05 versus the DMSO-treated cells of day 2. Student’s t-test was used to analyze the difference between the DMSO-treated and M5-treated cells of each time point. (**B**) Western blot analyses. Confluent C3H10T1/2 cells (U) were induced to undergo osteoblastic differentiation for 6h and 24 h at the presence of either DMSO or MCC-555 (5 μM). The signals of the Ser33/37/Thr41-phosphorylated β-catenin were quantitated and normalized to total β-catenin. All the normalized signals were compared to that of the untreated control (to which a value of 1 was assigned). Data represent the mean ± S.D. from three experiments. One-way ANOVA plus Scheffe’s post hoc tests were used to analyze the differences. ^*^, P<0.05 versus the untreated cells.

To further delineate the mechanism underlying MCC-555’s pro-osteogenic effect, we examined if MCC-555 was able to inactivate GSK3β given that GSK3β inactivation stabilizes cytoplasmic β-catenin for subsequent nuclear translocation. We induced C3H10T1/2 cells to undergo osteoblastic differentiation with or without MCC-555 co-treatment, and focused on the first 8 days to examine the Ser9-phosphorylation of GSK3β. As shown, the levels of Ser9-phosphorylated GSK3β in both groups largely decreased with time, however, MCC-555 elicited early induction of Ser9-phosphorylation (Figure 3A). The levels of Ser9-phosphorylated GSK3β in cells co-treated with MCC-555 at days 2, 4, 6, and 8 were 1.80 (P<0.01), 5.68 (P<0.05), 2.06 (P=0.061), and 1.32 (P=0.222) fold, respectively, of those of the counterpart control cells. These data indicated that MCC-555 was able to inhibit GSK3β kinase activity within 2 days. Subsequently, we focused on the first 24h and examined if MCC-555 co-treatment also decreased the phosphorylation of β-catenin at the Ser33/37/Thr41 sites. We induced cells to undergo osteoblastic differentiation, and treated cells with either DMSO or MCC-555 for 6h and 24h. Western blot analyses showed that MCC-555 decreased β-catenin phosphorylation. The levels of phosphorylated β-catenin in DMSO- and MCC-555-treated cells were 1.26 and 0.83 fold, respectively, of that of the untreated control at 6h, and were 1.30 and 0.65 (P<0.05) fold, respectively, of that of the untreated control at 24h (Figure 3B). Taken together, our data suggested that inhibition of GSK3β and stabilization of β-catenin might be a mechanism underlying the pro-osteogenic effect of MCC-555.

### FASN knockdown selectively inhibited the pro-adipogenic effect of MCC-555

**Figure 4:**
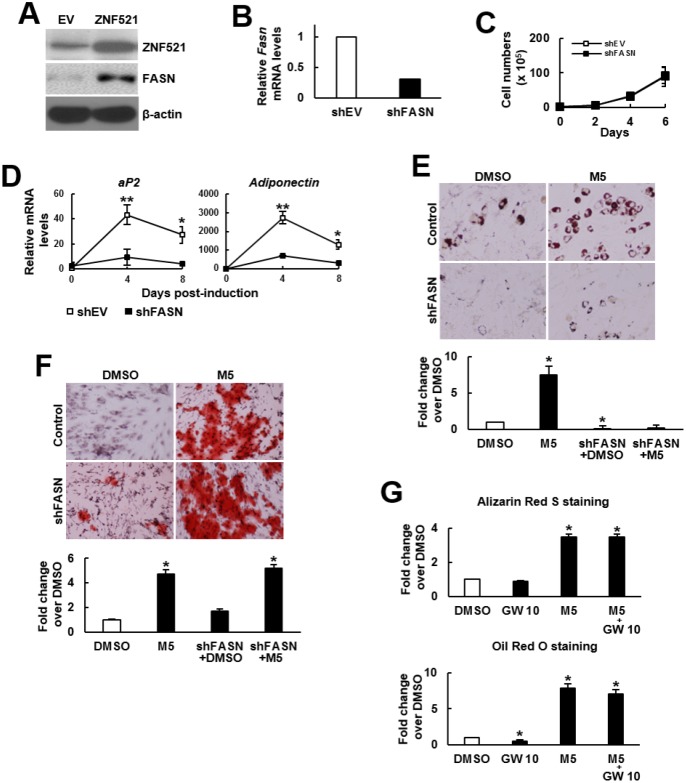
**Effect of MCC-555 plus FASN knockdown or GW9662 on the adipogenic and osteoblastic differentiation of C3H10T1/2 cells.** (**A**) Western blot analysis. ZNF521 overexpression induced FASN expression in C3H10T1/2 cells. (**B**) RT-qPCR analyses showed the *Fasn* mRNA levels of control (shEV) and FASN-knockdown (shFASN) cells. (**C**) Cell proliferation assays. shEV and shFASN cells were seeded (1 x 10^5^/plate) and counted at the time indicated. Data represent the mean ± S.D. from three experiments. (**D**) RT-qPCR analyses. Confluent shEV and shFASN cells were induced to undergo adipogenic differentiation, the expression kinetics of *aP2* and *adiponectin* mRNAs were examined at the times indicated. ^*^, P<10^-5^; ^**^, P<10^-6^ versus counterpart shEV control. (**E**) Adipogenic induction. Confluent shEV and shFASN cells were induced to undergo adipogenic differentiation. The cells were co-treated with 5 μM MCC-555 or DMSO in the first 3 days. Cells were stained with Oil Red O at the 8^th^ day. Representative photos are shown. The stains were quantitated, and the signals of the MCC-555-treated cells were compared to that of the DMSO control cells (to which a value of 1 was assigned). One-way ANOVA plus Scheffe’s post hoc tests were used to analyze the differences. ^*^, P<0.05 versus the DMSO-treated cells. (**F**) Osteoblastic induction. Confluent shEV and shFASN cells were induced to undergo osteoblastic differentiation. The shEV cells were co-treated with either 5 μM MCC-555 or DMSO, whereas the shFASN cells were co-treated with 5 μM MCC-555. Cells were stained with Alizarin Red S at the 28^th^ day. Representative photos are shown. The stains were quantitated, and the signals of the MCC-555-treated cells were compared to that of the DMSO control cells (to which a value of 1 was assigned). One-way ANOVA plus Scheffe’s post hoc tests were used to analyze the differences. ^*^, P<0.05 versus the DMSO-treated cells. (**G**) Confluent C3H10T1/2 cells were induced to undergo osteoblastic (upper) and adipogenic (lower) differentiation. Osteoblastic induction was accompanied by co-treated with DMSO, MCC-555 (5 μM), GW9662 (10 μM), or MCC-555 plus GW9662 (M5+GW10), whereas adipogenic induction was accompanied by those co-treatments in the first 3 days. Cells were stained with Oil Red O at the 8^th^ day or with Alizarin Red S at the 28^th^ day. The stains were quantitated, and the signals were compared to that of the DMSO control cells (to which a value of 1 was assigned respectively). One-way ANOVA plus Scheffe’s post hoc tests were used to analyze the differences. ^*^, P<0.05 versus the DMSO-treated cells.

The duel enhancing effect of MCC-555 and troglitazone inspired us to examine the possibility of selective inhibition of the pro-adipogenic effect of TZDs. We performed structure-and-reactivity examinations on MCC-555 and its derivatives, and found that MCC-555 might exert the duel enhancing effect via the same functional group (Supplementary Figure 1). This finding suggested that targeting on MCC-555 for the inhibition of its pro-adipogenic effect might inhibit its pro-osteoblastic effect, too. Since that MCC-555 is an inhibitor of ZNF521 expression, and that FASN is a critical effector of ZNF521’s pro-adipogenic activity, we tested the combination of MCC-555 with FASN knockdown for this task. Here we showed that ZNF521 overexpression induced FASN expression even without adipogenic induction (Figure 4A). We prepared FASN-knockdown (shFASN) C3H10T1/2 cells whose *Fasn* mRNA expression was 69% less than that of the control (shEV) cells (Figure 4B). Next, we examined the impact of FASN knockdown on cell proliferation given that proliferation rate determines the confluency of cell cultures and the intensity of differentiation. In the differentiation experiments, cells were cultured for 3 days to reach confluency for differentiation induction. Therefore, we counted the cell number of sub-confluent cultures every 2 days for up to 6 days. We found that FASN knockdown did not decrease or increase proliferation rate (Figure 4C). So then, the shEV and shFASN cells were induced to the adipogenic lineage for subsequent RT-qPCR analyses. The data showed that FASN knockdown repressed the induction of two adipocyte markers, aP2 and adiponectin (Figure 4D), indicating the inhibition of adipogenesis by FASN knockdown. Next, shEV and shFASN cells were induced to undergo adipogenic and osteoblastic differentiation with or without MCC-555 co-treatment. Our data showed that in the adipogenic induction group, the lipid droplet formation in the MCC-555-treated shEV cells was 7.5 fold (P<0.05) of that of the DMSO-treated shEV cells, however, this activity in DMSO-treated and MCC-555-treated shFASN cells was 0.04 (P<0.05) and 0.3 fold, respectively, of that of the DMSO-treated shEV cells (Figure 4E). In the osteoblastic induction group, the calcium precipitation activity of the MCC-555-treated shEV cells was 4.3 fold (P<0.05) of that of the DMSO-treated shEV cells, however, this activity of the DMSO-treated and MCC-555-treated shFASN cells were 1.7 and 5.2 fold (P<0.05), respectively, of that of the DMSO-treated shEV cells (Figure 4F). Our data showed that FASN knockdown selectively repressed the pro-adipogenic effect of MCC-555. On the other hand, FASN is an enzyme catalyzing the *de novo* synthesis of fatty acids from acetyl-CoA and malonyl-CoA. Fatty acids can act as PPARγ ligands to regulate the genes involved in glucose and lipid homeostasis. This led us to examine if a PPARγ2 inhibitor, GW9662, could selectively repress the pro-adipogenic effect of MCC-555. We treated C3H10T1/2 cells with MCC-555 (5 μM), GW9662 (10 μM), and MCC-555 plus GW9662, respectively, and induced cells to undergo osteoblastic and adipogenic differentiation. Data showed that compared to DMSO control, GW9662 seemed not to affect calcium precipitation, whereas MCC-555 and MCC-555 plus GW9662 both caused approximately 3.5 fold (P<0.05) increase in calcium precipitation (Figure 4G, upper). On the other hand, while GW9662 decreased 50% (P<0.05) of lipid droplet formation, it was not able to significantly attenuate the MCC-555-induced lipid droplet formation (Figure 4G, lower).

**Figure 5:**
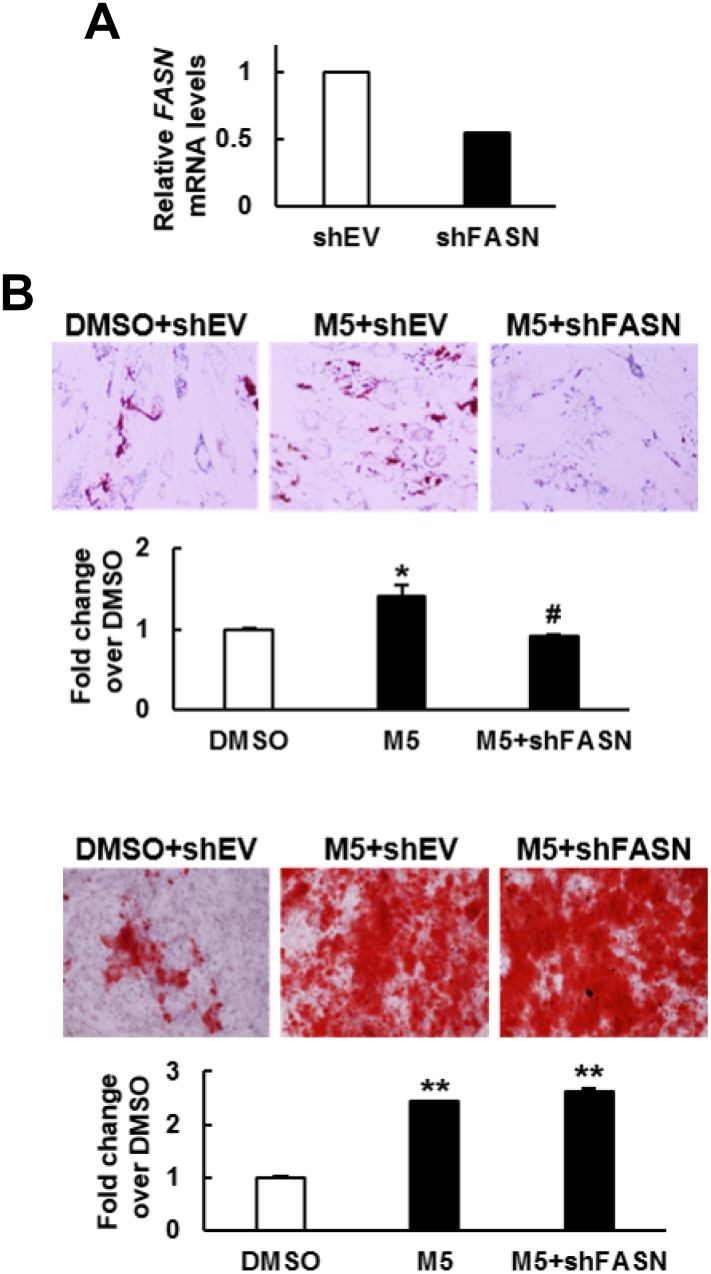
**Effect of MCC-555 plus FASN knockdown on the adipogenic and osteoblastic differentiation of human bmMSCs.** (**A**) RT-qPCR analyses showed the *FASN* mRNA levels of control (shEV) and FASN-knockdown (shFASN) cells. (**B**) Confluent shEV and shFASN cells were induced to undergo adipogenic (upper panel) and osteoblastic (lower panel) differentiation. The shEV cells were co-treated with either 5 μM MCC-555 or DMSO, whereas the shFASN cells were co-treated with 5 μM MCC-555. Cells were stained with Oil Red O at the 12^th^ day, and with Alizarin Red S at the 14^th^ day. Representative photos are shown. The stains were quantitated, and the signals of the MCC-555-treated cells were compared to that of the DMSO control cells (to which a value of 1 was assigned). ^*^, P<0.05; ^#^, P=0.73; ^**^, P<0.005 versus DMSO control.

To examine if the pro-osteoblastic effect of MCC-555 plus FASN knockdown seen in C3H10T1/2 cells also applied to human bmMSCs, we knocked down approximately 46% of the *FASN* mRNA expression in bmMSCs prepared from an aged human donor (Figure 5A), and induced cells to the adipogenic and osteoblastic lineage separately in the presence or absence of 5 μM of MCC-555. The data showed that the lipid droplet formation activity of the MCC-555-treated shEV cells were 1.4 fold (P<0.05) of that of the DMSO-treated shEV cells, however, MCC-555-treated shFASN cells were 0.9 fold (P=0.73) of that of the DMSO-treated shEV cells (Figure 5B, upper). The calcium precipitation activity of the MCC-555-treated shEV and shFASN cells were 2.4 (P<0.005) and 2.6 fold (P<0.005) of that of the DMSO-treated shEV cells (Figure 5B, lower). So, it was as consistent in human bmMSCs as in C3H10T1/2 cells that FASN knockdown selectively blocked the pro-adipogenic effect of MCC-555.

### Combination of MCC-555 and FASN knockdown improved osteogenic performance of aging human bmMSCs in a 3-dimentional scaffold cell culture system

**Figure 6:**
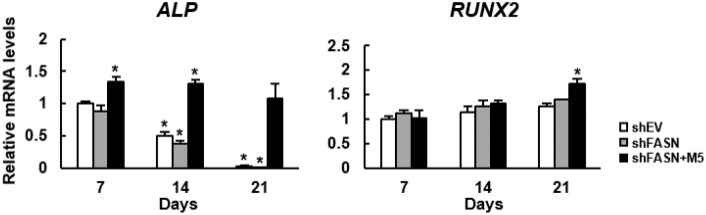
**Effect of MCC-555 to the osteoblastic differentiation of 3-dimentional bmMSC cultures.** Scaffolds which contained either control (shEV) or FASN-knockdown (shFASN) or FASN-knockdown plus MCC-555 (5 μM) co-treated (shFASN+M5) human bmMSCs were incubated under osteoblastic induction in the bioreactor, and retrieved at days 7, 14, and 21 for total RNA preparation. The levels of *RUNX2* and *ALP* mRNAs were measured by RT-qPCR analyses. Normalized signals were compared to those of shEV of day 7 (to which a value of 1 was assigned). Data represent the mean ± S.D. from three experiments. One-way ANOVA plus Scheffe’s post hoc tests were used to analyze the differences. ^*^, P<0.05 versus shEV cells of day 7.

Based on our data, we thought that we might have developed a strategy to turn MCC-555 into an enhancer of bone formation. So, we used a bioreactor to mimic *in vivo* condition to assess if MCC-555 could enhance the bone-forming capability of aging human bmMSCs. First, we seeded FASN-knockdown (shFASN) and corresponding control (shEV) human bmMSCs into calcium-alginate scaffolds separately, and induced cells to undergo osteoblastic differentiation separately in a constantly perfused bioreactor system. In particular, the scaffolds seeded with FASN-knockdown cells were divided into two groups, one of them was co-treated with 5 ¼M of MCC-555. The media and MCC-555 were not changed during the experiment, and the scaffolds were retrieved 7, 14, and 21 days after. We extracted RNAs from the scaffolds, and examined the expression of *alkaline phosphatase* (*ALP*) and *RUNX2* mRNAs (Figure 6). ALP is an osteoblast marker, and RUNX2 is the major transcriptional activator of osteoblastic differentiation. RT-qPCR analyses showed that the expression of *ALP* mRNA in shEV and shFASN cell clusters declined with time, whereas MCC-555 increased *ALP* expression at day 7 and sustained. The expression of *RUNX2* mRNA increased with time in three groups of cells, and MCC-555 co-treatment significantly (P<0.05) increased the expression at day 21. The up-regulation of *ALP* and *RUNX2* mRNAs indicated that MCC-555 enhanced osteoblastic differentiation in the 3-dimentional cultures.

**Figure 7:**
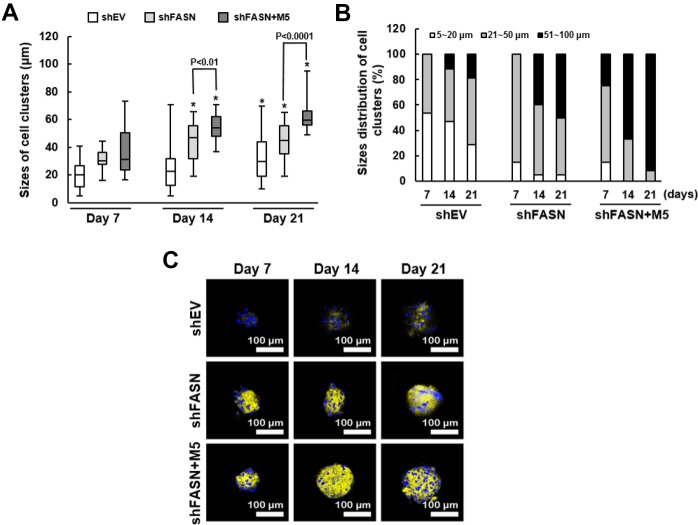
**Size distribution and biomineralization of bmMSC cell clusters.** (**A**) The sizes of cell clusters in the scaffolds incubated in the bioreactor for 7, 14, and 21 days were measured by SEM. One-way ANOVA plus Scheffe’s post hoc tests were used to analyze the differences. ^*^, P<0.05 versus shEV cells of day 7. Student’s t-test was used to analyze the difference between the shFASN and shFASN plus M5-treated cells of days 7, 14 and 21. (**B**) The cell clusters of shEV, shFASN, and shFASN+M5 groups were categorized by the size (5~20 μm, 21~50 μm, and 51~100 μm), and the percentage was calculated. (**C**) The cell clusters were stained with xylenol orange to indicate the biomineralized areas, and with Hoechst 33342 to indicate the location of nucleus.

Second, we analyzed the size distribution of the cell clusters using scanning electron microscopy (SEM). The mean size of clusters from shEV control, shFASN, and shFASN plus MCC-555 co-treatment groups were 19.4 μm (range, 5~41 μm), 31.1 μm (range, 16~44 μm), and 37.7 μm (range, 16~73 μm), respectively, at day 7; 24.9 μm (range, 5~71 μm), 44.1 μm (range, 19~66 μm), and 54.5 μm (range, 37~71 μm), respectively, at day 14; and 33.3 μm (range, 11~70 μm), 44.7 μm (range, 19~61 μm), and 62.9 μm (range, 49~95 μm), respectively, at day 21 (Figure 7A). These data showed that the aging bmMSCs were able to form clusters with increasing sizes under osteoblastic induction, and that this process was significantly (P<0.05) facilitated by FASN knockdown at days 14 and 21, which was further promoted by MCC-555 co-treatment (P<0.05). We also examined in each group the percentage of clusters with size around 5~20 μm, 21~50 μm, and 51~100 μm, respectively (Figure 7B). For shEV group, the percentage was 53.3%, 46.7%, and 0% at day 7; 47.1%, 41.2%, and 11.7% at day 14; and 28.6%, 52.4%, and 19% at day 21. For shFASN group, those were 15%, 85%, and 0% at day 7; 5%, 55%, and 40% at day 14; and 5%, 45%, and 50% at day 21. For shFASN plus MCC-555 co-treatment groups, those were 15%, 60%, and 25% at day 7; 0%, 33.3%, and 66.7% at day 14; and 0%, 8.3%, and 91.7% at day 21. These data also indicated that shFASN plus MCC-555 exhibited the strongest enhancing effect on the increase of cluster size under osteoblastic induction. In parallel, we analyzed the biomineralization status of the cell clusters. Our analyses showed that calcification of cell clusters in shFASN and shFASN plus MCC-555 co-treatment groups took place earlier and was stronger than in shEV control group, and that MCC-555 further intensify the calcification in the background of FASN knockdown (Figure 7C). Taken together, these data indicated that MCC-555 was able to enhance the bone-forming capability of aging human bmMSCs.

## DISCUSSION

We have conducted a “proof-of-concept” study to examine if the inhibition of the expression and the pro-adipogenic activity of ZNF521 can improve the osteogenic performance of bmMSCs derived from an aged human donor. While extensive efforts have been made to synthesize new TZDs with safer bone effect, our study of the regulatory role of troglitazone and MCC-555 on the lineage potentials of mouse and human multipotent cells has led to the discovery that TZDs are also, in fact, enhancers of osteogenesis. Informatively, the duel enhancing effect of MCC-555 on lipid droplet formation and calcium precipitation was not seen with the compounds {4-[(2-fluorophenyl)methoxy] phenyl}methanamine and 5-[6-(benzyloxy)naphthalen-2-yl]pyridine-2-ol, which are MCC-555 derivatives (Supplementary Figure 1). Thus, MCC-555, and perhaps all the TZDs, might exert this dual enhancing effect through the thiazolidinedione moiety.

While PPARγ is the major mediator of TZD’s pro-adipogenic effect, we have shown that TZDs, MCC-555 in particular, might target the canonical Wnt/β-catenin pathway to boost osteoblastic differentiation. The first line of evidence came from the examination of the nuclear translocation of β-catenin. The data that osteoblastic induction decreased nuclear β-catenin with time (Figure 2A), and did not cause significant calcium precipitation even in 24 days (Figure 2B), indicated in this study that it might take more than 20 days for our differentiation inducers to induce nuclear translocation of β-catenin and calcium precipitation. These processes, however, were facilitated and enhanced by MCC-555 co-treatment within 24 days under the same experimental setting (Figure 2). The second line of evidence came from the examination of the phosphorylation of GSK3β and β-catenin. Our data showed that MCC-555 increased the Ser9-phosphorylated GSK3β levels in 2 days (Figure 3A), indicating that MCC-555 might inhibit the GSK3β activity within 48h or even earlier after treatment. Indeed, the data that MCC-555 co-treatment decreased the phosphorylation of β-catenin at the Ser33/37/Thr41 sites within 24h (Figure 3B) supported this notion. It is currently unclear how the inhibition of GSK3β activity taking place in hours after MCC-555 treatment links to the nuclear translocation of β-catenin several taking place several days later. It is reasonable to speculate that MCC-555 had to counteract the inhibitory effect of osteoblastic induction on the nuclear translocation of β-catenin (Figure 2A), which might delay our observation of the MCC-555-induced increase in β-catenin translocation. Thus, our data suggest that MCC-555 might act as a Wnt ligand to inhibit GSK3β for subsequent stabilization and nuclear translocation of β-catenin.

Despite the *in vitro* duel enhancing effect, administration of TZDs, however, resulted in bone loss. To interpret these observations, it is noteworthy that in our study, it took more times for MCC-555 to enhance calcium precipitation than the time needed to enhance adipogenic differentiation (Figures 4 and 5). Thus, the pro-osteogenic effect of TZDs occurring at a later time point might be overwhelmed by their pro-adipogenic effect in the *in vivo* context. Accordingly, selective inhibition of the pro-adipogenic effect of TZDs is required for observing the pro-osteoblastic effect of TZDs. The finding that MCC-555 might exert its duel enhancing effect via its thiazolidinedione moiety suggests that it might be more practical to target something other than the thiazolidinedione moiety to selectively block the pro-adipogenic effect of TZDs. We show that FASN could be an ideal target because the combination of MCC-555 with FASN knockdown selectively blocked adipogenic differentiation of the 2-dimentional cultures (Figures 4 and 5), and improved bone-forming capability of the 3-dimentional cultures of aging human bmMSCs (Figures 6 and 7). The cell survival rate in the clusters of FASN-knockdown plus MCC-555 group was not higher than that of the FASN-knockdown group (Supplementary Figure 2), indicating that the MCC-555-induced increase of cluster size in scaffolds is due to increased calcification rather than increased cell proliferation. In this study, GW9662 (10 μM) inhibited adipogenic differentiation but failed to attenuate MCC-555-induced adipogenesis (Figure 4G). We have used higher dose of GW9662, and found that 20 μM GW9662 further inhibited adipogenic differentiation, but caused cell death during osteoblastic induction (data not shown). These data indicate that while the effectiveness of PPARγ2-targeted approach in the selective inhibition of the pro-adipogenic effect of MCC-555 is currently unclear, the FASN-targeted approach may be a plausible alternative for this task.

While our data depict a formula containing TZDs for boosting osteogenesis, a potential concern of using TZDs to treat osteoporosis and promote the healing from bone injury is that TZDs might concomitantly disrupt glucose uptake and energy metabolism given that TZDs are potent antidiabetic agents. However, our formula also contains FASN-targeted inhibition. It is worthy to note that FASN catalyzes the synthesis of fatty acids including those acting as PPARγ ligands, and FASN knockdown might, therefore, down-regulate PPARγ activity accordingly. While TZDs exert their antidiabetic effect through PPARγ, concomitant FASN-targeted inhibition might retain the pro-osteogenic effect of TZDs but counteract their antidiabetic effect. More investigations are required to support this notion. Importantly, our data indicate that MCC-555 combined with FASN knockdown may serve as an effective *in vitro* method to activate the osteogenic performance of aging bmMSCs, which paves the way for the clinical application of MCC-555 in the treatment of bone injury. First, for possible application of autologous bmMSCs in treating bone injuries of the elderly, our proposed combinational formula can be applied to activate the bone-forming capability of aging human autologous bmMSCs *in vitro* for limited *in situ* delivery to repair local bone damage. Second, for the treatment of large scale bone injuries of the elderly patients, our proposed combinational formula can facilitate massive production of bone-like tissues by aging bmMSCs in the bioreactor. Those tissues can be disinfected and used as bone graft materials.

In conclusion, we have disclosed that TZDs can potentially act as a GSK3β inhibitor to enhance osteogenesis, that TZDs’ pro-adipogenic effect can be selectively blocked, and most importantly that TZDs can be used to activate the osteogenic performance of aging bmMSCs for therapeutic purposes. Our data point out the necessity of searching for the pharmacological agents that inhibit adipogenic differentiation of bmMSCs in a way compatable to that of FASN knockdown. The scenario displayed here is driven by the concept of combining the inhibitors of the expression and the pro-adipogenic activity of ZNF521. In this regard, the role of ZNF521 as a promoter of skeletal aging, and as an intervention target in the screening for pharmacological agents that enhance bone formation is suggested.

## MATERIALS AND METHODS

### Chemicals and antibodies

MCC-555, Oil Red O, Alizarin Red S, GW9662, and Cetylpyridinium chloride (CPC) were purchased from Sigma-Aldrich (MO, USA). {4-[(2-fluorophenyl) methoxy]phenyl}methanamine and 5-[6-(benzyloxy) naphthalen-2-yl]pyridine-2-ol were purchased from HDH Pharma Inc., (NC, USA). Sodium alginate was purchased from FMC Health and Nutrition, (PA, USA). Antibodies against β-catenin, Ser33/37/Thr41-phosphorylated β-catenin, GSK3β, Ser9-phosphorylated GSK3β, Lamin B, and β-actin were purchased from Cell Signaling Technology, (MA, USA).

### 2-dimentional cell culture and induction of differentiation

C3H10T1/2 cells were purchased from American Type Culture Collection, and were maintained in DMEM (Thermo Fisher Scientific, MA, USA). Human bmMSCs were isolated as described previously [16] from bone marrows collected from a 64-year old male patient with informed consent. The use of human bmMSCs was approved by the Institutional Review Board of National Taiwan University Hospital, Hsin-Chu Branch (IRB No. 103-012-F). BmMSCs were maintained in Dulbecco's modified Eagle's medium (DMEM) (low glucose) (Thermo Fisher Scientific, MA, USA) containing fetal bovine serum (15%) (Hyclone), glutamine, penicillin and streptomycin. Both C3H10T1/2 cells and human bmMSCs were maintained in a humidified atmosphere containing 5% CO_2_ at 37°C. Cell culture media were changed every 4 days. The induction of adipogenic and osteoblastic differentiation of C3H10T1/2 cells and human bmMSCs was performed as described previously [17]. Briefly, for adipogenic induction, confluent C3H10T1/2 cultures were maintained in medium plus dexamethasone, isobutylmehtylxanthine, and insulin for 3 days, then in medium plus insulin for 2 days, and then in culture medium for 3 days. Confluent human bmMSCs were subjected to runs of the treatment as described for C3H10T1/2 cells until the oil droplets were seen. For osteoblastic induction, confluent C3H10T1/2 cultures and human bmMSCs were maintained in medium plus dexamethasone, ascorbic acid 2-phosphate, and glycerol 2-phosphate until the end of the experiment, with medium changed every three days. At the end of experiments, cells induced to the adipocyte lineage were fixed and stained with 0.3% Oil Red O, and were de-stained with isopropanol for the measurement of absorbance at 510 nm, whereas cells induced to the osteoblast lineage were fixed and stained with 2% Alizarin Red S solution and then de-stained with freshly prepared 10% cetylpyridinium chloride (CPC) (Sigma-Aldrich, MO, USA) solution. The CPC solutions were then collected for the measurement of absorbance at 595 nm.

### Plasmids, Lentivirus preparation, and infection

Plasmid harboring human ZNF521 complementary DNA (pLAS2w-ZNF521) was constructed as described [17]. Plasmids harboring shRNA targeting either human *FASN* mRNA (pshRNA_FASN_, clone ID:TRCN0000003126) or mouse *Fasn* mRNA (pshRNA_Fasn_, clone ID:TRCN0000075703) were purchased from the National RNAi Core Facility at Academia Sinica, Taiwan. These plasmids were co-transfected with gag-pol and VSV-G-expressing plasmids into 293T cells. Viral supernatant collected 24, 48, and 72 h post-transfection was pooled and filtered through 0.45-μm filters. For infection, cells were infected with virus (MOI = 40) for 3 h in the presence of polybrene (8 μg/ml), and then maintained in regular medium.

### Quantitative real-time PCR (RT-qPCR) and Western blot analyses

Total RNA of 2-dimentional cultures and cells growing inside the scaffolds was isolated as described previously using TriRNA pure kit [29]. Total RNA was subjected to reverse transcription-PCRs to generate complimentary DNAs. RT-qPCR was performed as described [16]. The 5’ and 3’ primers used were as follows: mouse *Pparγ2*, TCGCTGATGCACTGCCTATG and GAGAGGTCCACAGAGCTGATT; mouse *Zfp521*, GAAACCGAGATCCCTCAAAGA and TCTGGCCTCTTCTTGCAGTC; mouse *Fasn*, CCTGCCCAATCTCTATAGTGTCTCTAC and AAGGTTTTATTGCCTCTCATCCAT; mouse *osteopontin*, CCATCTCAGAAGCAGAATCTCC and ATGGTCATCATCGTCGTCC; mouse *osteocalcin*, TCTCTCTGACCTCACAGATCCC and TACCTTATTGCCCTCCTGCTTG; mouse *β-actin*, CCCTGGCACCCAGCAC and GCCGATCCACACGGAGTAC; human *FASN*, GCAAATTCGACCTTTCTCAGAAC and GGACCCCGTGGAATGTCA; human *ALP*, GACCCTTGACCCCCACAAT and GCTCGTACTGCATGTCCCCT; human *RUNX2*, TTTGCACTGGGTCATGTGTT and TGGCTGCATTGAAAAGACTG; and human *β-actin*, AAGTCCCTTGCCATCCTAAAA and ATGCTATCACCTCCCCTGTG. The relative mRNA levels were calculated using the 2^-Δ Δ CT^ method, with *β-actin* mRNA as a normalizer.

### 3-dimentional culturing of human bmMSCs in a continuously perfused bioreactor system

The preparation of calcium-alginate scaffolds, seeding of bmMSCs into scaffolds, assembling of a bioreactor system, and culturing of the scaffolds in bioreactor were performed as described previously [27–29]. Briefly, calcium-alginate was first frozen at −20°C overnight and then freeze-dried to form porous structure. Subsequently, the porous scaffolds were cross-linked in 5% calcium chloride solution twice, sterilized with 75% alcohol, and stored at room temperature. Human bmMSCs were transferred into scaffolds (5 × 10^5^ viable cells/scaffold) by injection, maintained in a non-coating culture plate for 24 h. Then, 10 scaffolds were transferred into the cell tank of the bioreactor system. Each cell tank was connected with a medium tank containing 500 ml medium supplemented with 20% FBS. MCC-555 and osteogenic inducers were added in the medium tank at day 0. The scaffolds were cultured in the bioreactor for 7, 14, and 21 days without changing the medium. The bioreactor system contained a peristaltic pump to generate medium flow within the system to replenish nutrition and oxygen supply to the scaffolds.

### Scanning electron microscopy (SEM) and size distribution analysis of human bmMSC clusters

For size distribution analysis of human bmMSC clusters, scaffolds were retrieved from the bioreactor at the time indicated. The scaffolds were fixed, dehydrated, dried, and coated with gold before observation as described previously [29], and the morphology of human bmMSC clusters was imaged with SEM (TM-1000, Hitachi, Japan). The diameters of the clusters were calculated with MetaMorph Microscopy Automation and Image Analysis Software (Molecular Devices, CA, USA). We analyzed 15 to 24 cell clusters for each condition in this study.

### Examination of cell survival rate and biomineralization of the cell clusters

The scaffolds retrieved from the bioreactor were stained with 4 μM of calcein AM (Life Technologies Inc., CA, USA), and then with 4 μM of propidium iodide (PI, Life Technologies, Inc., USA). Live cells showed green fluorescence (ex/em ~495 nm/~515 nm), whereas dead cells showed red fluorescence (ex/em ~540 nm/~615 nm). Cell survival rate was estimated with a confocal microscope (LSM 780, Zeiss, Germany), and the 3-dimentional images were reconstructed using the supplied software (ZEN lite, Zeiss, Germany). Subsequently, xylenol orange staining was used to examine the biomineralization of the cell clusters inside the scaffolds. The scaffolds were fixed with 4% para-formaldehyde, stained with 20 μM xylenol orange (Sigma-Aldrich, MO, USA), and counterstained with 1 μg/ml Hoechst 33342 (Sigma-Aldrich, MO, USA), and observed with a confocal microscope as described previously [29]. The biomineralized areas of the cell clusters stained by xylenol orange showed bright orange-red (ex/em ~440 nm/~610 nm), whereas the nucleus stained by Hoechst 33342 showed blue color. The 3-dimentional images were reconstructed using the supplied software.

### Statistical analysis

Data were analyzed by one-way ANOVA followed by Scheffe’s post hoc tests as indicated. Otherwise, Student’s t-test was used for data analyses. P value less than 0.05 was considered statistically significant.

## Supplementary Material

Supplementary Figures
