# Supplementary material for: Cooperative impact of thiazolidinedione and fatty acid synthase on human osteogenesis
Source: Aging (Albany NY). 2019 Apr 20;11(8):2327–42. doi: 10.18632/aging.101916 (PMC6519991; doi:10.18632/aging.101916)
Supplement: Supplementary Figures [file aging-11-101916-s001.pdf]

SUPPLEMENTARY FIGURES

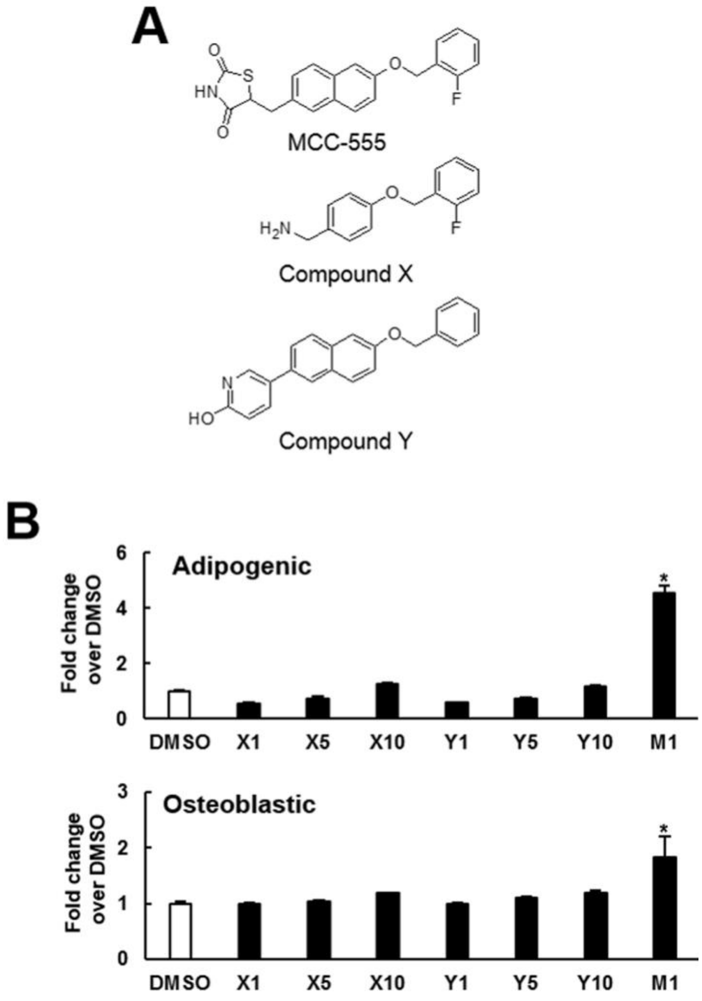

Supplementary Figure 1. Effect of MCC-555, and two MCC-555-related compounds on the adipogenic and osteoblastic differentiation of C3H10T1/2 cells. (**A**) Chemical structures of MCC-555, {4-[(2-fluorophenyl)methoxy]phenyl}methanamine (compound X), and 5-[6-(benzyloxy)naphthalen-2-yl]pyridine-2-ol (compound Y) are shown. (**B**) Adipogenic and osteoblastic differentiation. Confluent C3H10T1/2 cells were co-treated with DMSO (DMSO) or with 1, 5, and 10 μM compounds X or Y (X1, X5, X10, Y1, Y5, Y10), or with 1 μM MCC-555 (M1) in the first 3 days while under adipogenic induction. The same co-treatment was last for 28 days while cells were under osteoblastic induction. Cells were stained with Oil Red O at the 8th day for adipogenic differentiation, and with Alizarin Red S at the 28th day for osteoblastic differentiation. The stains were quantitated, and the signals of the MCC-555-, compound X- and compound Y-treated cells were compared to that of the DMSO-treated cells (to which a value of 1 was assigned). Data represents mean ± S.D. from three experiments. One-way ANOVA plus Scheffe's post hoc tests were used to analyze the differences. *, $P<0.05$ versus DMSO-treated cells.

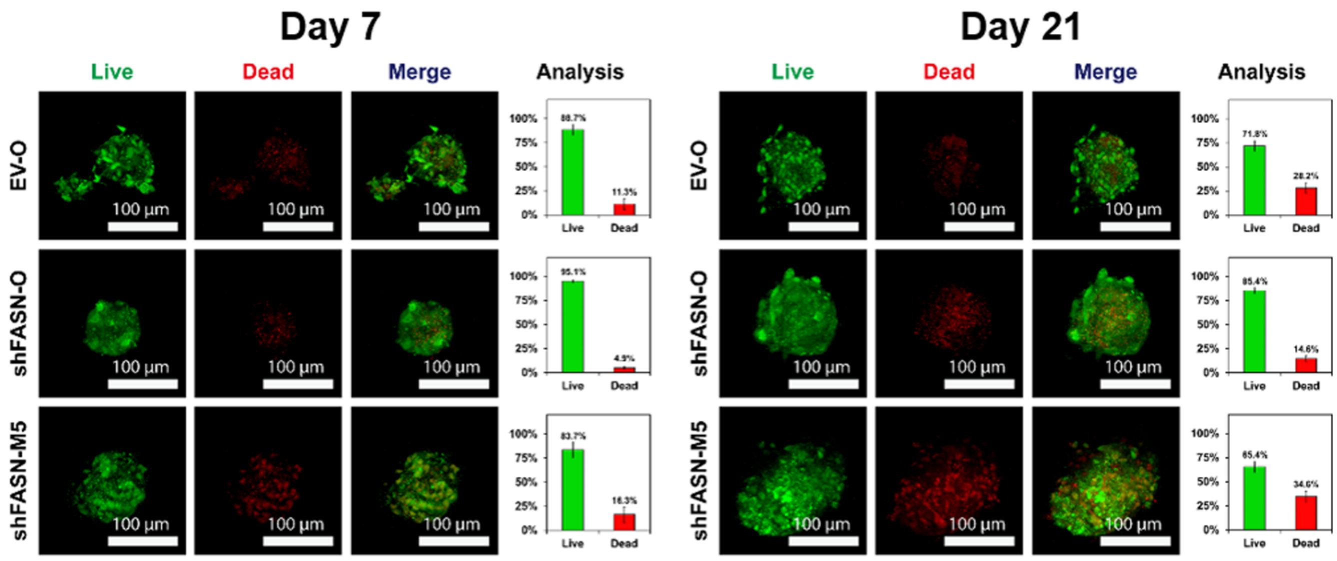

Supplementary Figure 2. Live/dead analyses on the cell clusters harvested from bioreactor. The cell-containing scaffolds cultured in the bioreactor for 7 and 21 days were harvested and processed for estimating the cell survival rates as described in the Materials and Methods. As shown, the cell survival rate of control (EV-O), FASN-knockdown (shFASN-O), and FASN-knockdown plus MCC-555 treatment (shFASN-M5-O) groups were approximately 88.7%, 95.1%, and 83.7%, respectively, at day 7, and were approximately 71.8%, 85.4%, and 65.4%, respectively, at day 21.
